# *In Vivo* Fate Imaging of Intracerebral Stem Cell Grafts in Mouse Brain

**DOI:** 10.1371/journal.pone.0144262

**Published:** 2015-12-07

**Authors:** Annette Tennstaedt, Alfonso Mastropietro, Melanie Nelles, Andreas Beyrau, Mathias Hoehn

**Affiliations:** 1 In-vivo-NMR Laboratory, Max Planck Institute for Metabolism Research, Cologne, Germany; 2 Scientific Direction Unit, IRCCS Foundation Neurological Institute “C. Besta”, Milan, Italy; 3 Politecnico di Milano, Department of Electronic Information and Bioengineering, Milan, Italy; 4 Department Radiology, Leiden University Medical Center, Leiden University, Leiden, Netherlands; Fraunhofer Institute for Cell Therapy and Immunology, GERMANY

## Abstract

We generated transgenic human neural stem cells (hNSCs) stably expressing the reporter genes Luciferase for bioluminescence imaging (BLI) and GFP for fluorescence imaging, for multimodal imaging investigations. These transgenic hNSCs were further labeled with a clinically approved perfluoropolyether to perform parallel ^19^F MRI studies. I*n vitro* validation demonstrated normal cell proliferation and differentiation of the transgenic and additionally labeled hNSCs, closely the same as the wild type cell line, making them suitable for *in vivo* application. Labeled and unlabeled transgenic hNSCs were implanted into the striatum of mouse brain. The time profile of their cell fate after intracerebral grafting was monitored during nine days following implantation with our multimodal imaging approach, assessing both functional and anatomical condition. The ^19^F MRI demarcated the graft location and permitted to estimate the cell number in the graft. BLI showed a pronounce cell loss during this monitoring period, indicated by the decrease of the viability signal. The *in vivo* obtained cell fate results were further validated and confirmed by immunohistochemistry. We could show that the surviving cells of the graft continued to differentiate into early neurons, while the severe cell loss could be explained by an inflammatory reaction to the graft, showing the graft being surrounded by activated microglia and macrophages. These results are different from earlier cell survival studies of our group where we had implanted the identical cells into the same mouse strain but in the cortex and not in the striatum. The cortical transplanted cells did not show any loss in viability but only pronounced and continuous neuronal differentiation.

## Introduction

Stem cell therapy is gaining a growing interest in medical research in recent years. The main goal is to repair and recover the damaged tissue by transplanting stem cells to replace the lost tissue/cells. The transplanted, differentiated stem cells are expected to promote cell repair of the damaged tissue and replace the lost tissue by integrating into the endogenous tissue, thereby recovering the lost or impaired functions [1, 2]. In particular, transplantation of neural stem cells (NSCs) is emerging as a treatment for e.g. neurological diseases such as neurodegeneration, stroke or other cerebral diseases [3]. However, important challenges still exist concerning a better understanding of the engraftment, viability, and safety behavior of transplanted stem cells, as well as their interaction with the milieu. Noninvasive molecular imaging techniques are a powerful tool to investigate the fate and the ultimate feasibility of stem cell transplantation therapy. Here, magnetic resonance imaging (MRI) plays an important role thanks to i) high spatial resolution, ii) non-invasiveness, and iii) unlimited tissue penetration. The application of superparamagnetic iron oxide (SPIO) particles was widely evaluated for labeling NSCs [4–6] in preclinical studies but this approach can lead to ambiguous interpretation due to the signal from the surrounding tissues, e.g. due to microbleedings. Furthermore, the iron from cells undergoing apoptosis or cell lysis can be internalized by microglia or macrophages surrounding the grafted stem cells, resulting in signal falsely attributed to cells [7].

Fluorine-19 (^19^F) MRI minimizes the problem of signal interpretation ambiguity, thanks to the absence of background signal from the tissue. ^19^F MRI allows direct detection of labeled cells for unambiguous identification and quantification. This imaging technique is gaining an increasing success in the last few years in the field of molecular imaging. Numerous applications for *in vivo* cell tracking have been reported in the literature and recent developments have brought ^19^F imaging technology closer to clinical application [8–10]. It should be noted, however, that the sensitivity of 19F MRI is clearly lower compared to T_2_*-weighted MRI of iron oxide labeled cells. T2*-weighted MRI of SPIO-labeled cells allows detection of individual cells under ideal conditions. Detection limit of 200 to 1.000 19F-labeled cells has been reported, as listed in a comprehensive review [9] which may be considered an impressively small group of cells for which preclinical ^19^F MRI studies have yielded very promising results [11, 12].

MRI generates the best anatomical localization of the cell graft but lack information about viability or functional state of transplanted NSCs. Therefore, progress comes from a multimodal imaging approach, which combines anatomical, morphological and functional information by using two or more imaging techniques [13]. Bioluminescence Imaging (BLI) has the high advantage to repetitively noninvasively monitor biologic phenomena *in vivo*, providing fast and effective ways for validating cell culture findings [14]. The photons of the BLI signal are generated by the oxidation of the substrate luciferin by the enzyme luciferase, expressed in transgenic cells. As luciferase is expressed solely in transgenic cells, there is no BLI background signal from residual tissue. Furthermore, an important aspect of this imaging modality, BLI monitors the viability and the fate of transplanted NSCs *in vivo*, as demonstrated in several studies of stem cell implantation into brain [15, 16] or spinal cord [17].

In the present investigation, we generated transgenic human NSCs which stably express the reporter genes for multimodal imaging Luc2 for BLI, and GFP for fluorescence imaging (FLI). We combined this strategy with labeling of hNSCs with a clinically approved perfluoropolyether (PFPE) preparation to perform parallel ^19^F MRI studies. These transgenic human cell lines were validated *in vitro* and applied *in vivo* in a longitudinal study after transplantation in the striatum of mouse brain. The time profile of the cell fate after intracerebral grafting was monitored with our multimodal imaging approach assessing both functional and anatomical condition. The *in vivo* obtained cell fate results were further validated and confirmed by immunohistochemistry. We show that the hNCS have a striatum specific vitality pattern quite different from that found in the cortex for the same cells [16].

## Materials and Methods

### Culturing of human NSCs

The human neural stem cell line (Life Technologies, Darmstadt, Germany), initially derived from the NIH-approved human embryonic stem cell line H9 (WA09) was used in this study and is termed in the following H9 hNSCs [18–20]. The H9 hNSCs were maintained according to the manufacturer´s protocol as monolayer culture on Geltrex coating at a density of 5x10^4^ cells/cm^2^ in StemPro NSC SFM complete medium consisting of 1x KnockOut DMEM/F-12, 2 mM GlutaMax, 20 ng/ml bFGF and EGF and 2% StemPro supplement (Life Technologies). The medium was changed every 2 days and every 3–4 days the cells were detached with accutase (PAA, Pasching, Austria).

### Generation of viral vector and transduction of H9 hNSCs

**Fig 1:**
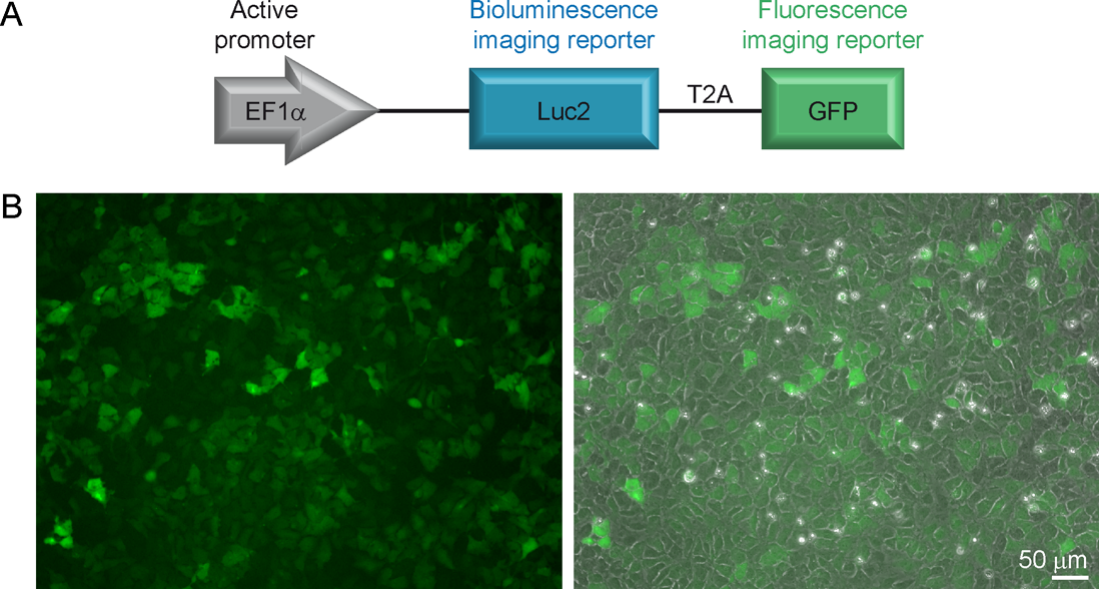
Newly generated hNSCs. (A) Schematic representation of the designed vector system. The two imaging reporters Luciferase 2 (Luc2) and green fluorescence protein (GFP) are kept under the control of the constitutive active promoter EF1α and are linked via the T2A peptide sequence to ensure equal expression level of the two proteins. (B) Representative microscopic image of transduced and FACS sorted hNSCs. The overlay of the bright-field and fluorescence image is shown right. Scale bar: 50 μm

The lentiviral backbone pCDH-EF1α-MCS-T2A-copGFP (System Biosciences, Mountain View, USA) was used in this study. The plasmid contains the human elongation factor 1 alpha (EF1α) promoter and the self-cleaving T2A sequence for efficient overexpression of two reporter genes. For the study, the plasmid was designed with the codon optimized firefly luciferase 2 (Luc2) from *Photinus pyralis* (Promega, Madison, USA) and the enhanced green fluorescent protein (copGFP = GFP) from *Pontenilla plumata* as the two imaging reporters. To clone Luc2 in the multiple cloning side (MCS) the following primer pair was used AAGGGAAAGGATCCGCCACCATGGAAGATCGCCAAAAACATTAAG (forward) and AAATTTGCGGCCGCCACGGCGATCTTGC (reverse) to generate pCDH-EF1α-Luc2-T2A-GFP. A schematic representation of the construct is displayed in Fig 1A.

For pseudoviral particle production 293T cells were transiently transfected with the HIV-lentiviral vector expression system, consisting of the generated pCDH plasmid containing viral genetic elements, and the packaging plasmids pPACKH1-GAG, pPACKH1-REV and pVSV-G providing the structural replication and integration proteins (System Biosciences). Briefly, 293T cells (2 x 10^6^) were seeded in T25 flasks (25 cm^2^) with DMEM and 10% FBS. After 24h cells were transfected with 2.25 μg pPACKH1 packaging plasmid mix and 1 μg target plasmid in Optimem (Life Technologies). The next day cells were washed and Optimem medium was added. The cells were incubated at 32°C and 5% CO_2_ for optimal virus generation. Supernatant was harvested 48h and 72h after transfection and centrifuged (1250 rpm for 10 min). The supernatant was used for lentiviral infection of the H9 hNSCs facilitated by addition of 8 μg/ml Polybrene (Sigma-Aldrich, Munich, Germany). Transduced H9 hNSCs were sorted by GFP expression with a fluorescence-activated cell sorting (FACS; FACS Canto, Becton Dickson, Franklin Lakes, USA) for high levels of GFP (10^3^−10^4^ counts). For sake of clarity we will name the new generated cell line “H9-EF1-Luc2-GFP”. Cell viability of transduced and non-transduced cells was analyzed by repetitive counting of viable cells with the trypan blue exclusion assay (mean of 6–8 passages). The influence of transduction of cell proliferation was checked during 5 passages and normalized to the wild type (WT) cells.

### Cell labeling

H9-EF1-Luc2-GFP cells were plated 4 hours before labeling (7.5*10^5^ cells per well for ^19^F labeling procedure and 6*10^5^ cells per well for unlabeled cells) in 6-well plates. 25 μl/ml PFPE nanoemulsion (Celsense-1000 (CS-1000), Celsense, Pittsburgh, USA) was added to the confluent cell layer and incubated for 42h. After the incubation time, cell viability/survival were determined by trypan blue asssay and proliferation was determined. Labeled cells were harvested and carefully washed two times with PBS to remove label excess, and quantity was adjusted to 1 x 10^5^ cells/μl in Hank´s buffered salt solution (HBSS) buffer (Gibco, Grand Island, NY, USA).

### Animal experimental protocol

All experiments were conducted according to the guidelines laid out in the German Animal Welfare Act and approved by the local authorities (Office for Nature, Environment and Consumer Protection North Rhine-Westphalia, Germany). In 13 adult male Nu/Nu mice, 1 x 10^5^ H9-EF1-Luc2-GFP cells were implanted in the right striatum while in the left striatum a sham injection was performed. Nine animals received cells labeled with ^19^F and 4 animals received unlabeled cells. The experimental protocol was composed of bioluminescence imaging (BLI) (day 0, 1, 2, 5, 7 and 9 post implantation) and ^19^F MRI (day 2 and 8 post implantation). The repetitive imaging procedures at such short intervals was stressful for the animals. Therefore, all animals were followed only for 9 days after stem cell implantation when a bioluminescence signal of viable cells was no longer detectable. Animals were housed in cages under a 12 h light/12 h darkness cycle with access to food and water ad libitum.

### Implantation procedure

At the day of implantation, 1 x 10^5^ labeled (n = 9) or unlabeled (n = 4) H9-EF1-Luc2-GFP cells were resuspended in 2 μl HBSS and kept on ice during surgery. Mice were anesthetized with 1–2% isoflurane in a 30/70 oxygen/nitrous oxide mixture, and 4mg/kg Carprofen (Pfizer, Berlin, Germany) was injected s.c. for analgesia. Each animal was fixed in a stereotactic frame (Stoelting, Dublin, Ireland) and the body temperature was maintained at 37°C (Medres, Cologne, Germany). The skin above the skull was cut with a small incision and a hole in the skull was drilled for implantation. The following coordinates were used for implantation into the striatum: AP: +0.5, ML: ± 2.0 mm from bregma, and DV: -3.0 mm from the brain surface. 100,000 cells were injected into the brain over a period of 5 min using a Hamilton syringe (26G needle) and a micropump system. After the deposit, the needle was kept in place for further 5 min before slow withdrawal. The wound was closed with suture and the animals recovered in a temperature-controlled cage.

### Bioluminescence imaging

*In vitro* experiments were performed with a photon imager (Biospace Lab, Paris, France) using the Photo Acquisition Software (version 2.7.5.1, 2008 BioSpace) and *in vivo* experiments were recorded with an IVIS SpectrumCT system (Perkin Elmer, Massachusetts, USA).

For *in vitro* BLI, 6 dilution series of ^19^F-labeled and unlabeled hNSCs (1.6*10^4^, 8*10^3^, 4*10^3^, 2*10^3^, 1*10^3^, 5*10^2^, 2.5*10^2^ cells) were used. Experiments were performed for both labeled and unlabeled H9-EF1-Luc2-GFP cells. hNSCs were seeded in a 96-well plate and immediately after the substrate administration (1 mM D-luciferin potassium salt dissolved in PBS; Synchem, Altenburg, Germany) acquisitions were carried out for 1h.

For *in vivo* BLI, mice were injected i.p. with 300 mg/kg luciferin (D-luciferin in potassium salt 99%, Synchem), and anesthetized with 3% isoflurane, following an earlier reported protocol [21]. Images were acquired every 5 min after substrate injection, for 30 min. The animals were placed on a 37°C degree heated holder. During acquisition the isoflurane was administered through a facial mask and the level was lowered to 2%.

### ^19^F MRI acquisitions

^19^F MRI and ^19^F magnetic resonance spectroscopy (MRS) acquisitions were carried out on a Biospec 11.7T/16 cm dedicated animal scanner (Bruker BioSpin, Ettlingen, Germany) equipped with actively shielded gradient coils (BGA9S, 750 mT m^-1^, Bruker BioSpin). For radiofrequency transmission and reception, we used a custom-built, inductively coupled, single-loop surface coil of 9 mm diameter for *in vitro* ^19^F MRS/MRI and a 20 mm diameter coil for *in vivo* MRI, all tunable from 470 MHz for the ^19^F resonance frequency up to 500 MHz for ^1^H imaging.

#### In vitro ^19^F MRS

In order to permit quantification of ^19^F load per cell, after the labeling procedure, ^19^F MRS was carried out on phantoms containing ^19^F labeled hNSCs. A single pulse spectroscopic sequence with short acquisition delay of 0.05 ms, and a repetition time (TR) of 20 s was used (90° rectangular hard pulse, duration/bandwidth (BW) = 0.01 ms/128 kHz, 163.8 ms acquisition window, spectral points/BW = 8192/50 kHz). The number of averages (NA) was 30, leading to an acquisition time (TA) = 10 min. As external reference, 10 μl of potassium fluoride (KF) at a concentration of 10 mg/mL was added to the sample tube containing the ^19^F labeled hNSCs.

#### In vivo ^19^F MRI

Mice were anesthetized with an intraperitoneal injection of a ketamine (100 mg/kg) and xylazine (10 mg/kg) mixture. After positioning, two catheters were connected to syringes containing ketamine (10 mg/ml) and xylazine (2 mg/ml), respectively. Forty min after the initiation of anesthesia, animals were re-infused at partial dosages every 20 min (for healthy animals, 25 mg/kg ketamine and 2.5 mg/kg xylazine). Respiration rate was monitored using a pneumatic sensor connected with the DASYlab (Measurement Computing, Norton, USA) software. The body temperature was maintained at 37°C with an in-house feedback controlled system. An external reference containing 500 μl of PFPE nanoemulsion in agar 1.5% at a concentration of 0.2 M ^19^F was placed at the side of the mouse forehead. Animals were scanned with ^1^H MRI, ^19^F MRS, and ^19^F MRI. The total time of the imaging session did not exceed 1.5 h.

Anatomical ^1^H imaging was performed with a turbo spin echo sequence (TR/effective echo time (TE_eff_) = 2200 ms/42.8 ms, 8 echoes per excitation, NA = 4, 16 consecutive, 0.5 mm thick slices, FOV = 2.88*1.92 cm^2^, 192*128 matrix, i.e. a resolution of 150*150*500 μm^3^, TA = 2 min 20 s, BW = 50 kHz, linear phase encoding scheme).

For correction of B_1_ inhomogeneities, 2 FLASH images at different flipangles (75° and 150°) were recorded, as suggested in previous work [22]. Images were acquired with the following parameters: TR = 4000 ms, TE = 3.106 ms, NA = 1, 8 consecutive, 1 mm thick slices, FOV = 2.88*1.92 cm^2^, 48*32 matrix, i.e. a resolution of 600*600*1000 μm^3^, TA = 2 min 8 s, BW = 100 kHz.

^19^F images were acquired with a RARE sequence at the following parameters: TR = 800 ms, TE = 10.5 ms, NA = 1120, TA = 59 min 44 sec, BW = 15 kHz. The geometry of the RARE images used the same geometry as for the FLASH images used for B_1_ inhomogeneities correction.

### Image and data analysis

MRI image analysis: All MR images were analyzed in Matlab (Mathworks, Inc., USA). For B_1_ correction, flip angle maps and ^19^F attenuation maps were calculated from the two FLASH images (neglecting 5% of all pixels with lowest signal values in both images). The noise in the ^19^F MR images was calculated using the histogram-based method described by Sijbers et al [23] and signal-to-noise (SNR) maps were calculated with a correction for low SNR in magnitude images as described by Gudbjardsson et al [24]. B_1_ corrected SNR maps were calculated by dividing the SNR map by the ^19^F attenuation map. Finally, cell density was calculated voxel-wise using the linear relationship:

$$\frac{Cells}{voxel}=\frac{SNR(voxel)}{SNR(reference)}∙\frac{19F\;per\;voxel}{19F\;per\;cell}$$

BLI image processing and analysis: Three regions of interest were drawn in each image, on the brain, on the body, and on the noise outside the body, in order to evaluate Signal to Background Ratio (SBR).

Signal to Background Ratio (SBR) was calculated voxel-wise using the following relationship:

$$SBR=\frac{\mu (Signal)-\mu (Background)}{\sigma (Noise)}$$

Where *μ(Signal)* is the mean flux in the ROI underlying the implantation site, *μ(Background)* is the mean value of ROI representing the background and *σ(Noise)* is the standard deviation of the ROI representing the noise. SBR was calculated longitudinally in each animal and normalized with respect to the first time point.

### Immunohistochemistry

For immunohistochemistry (IHC) the animals were deeply anesthetized with isoflurane and sacrificed. The brain was perfused, extracted and incubated in PFA 4% for 24h and then transferred in 30% sucrose solution for 48h. The brains were frozen and stored at -80°C. Brains were kryosectioned with a slice thickness of 14 μm and stored at -20°C.

In order to detect luciferase, luciferase antibody (Fitzgerald, Acton, USA,1:200) was added and conjugated with Cy3 donkey anti rabbit (1:200). For hNSCs detection, Human Nuclei antibody (Millipore, Billerica, USA, 1:100) was added and conjugated with Cy5 donkey anti mouse (1:200) or human Mitochondria antibody (MTC02, Dianova, Hamburg, Germany, 1:200) was used and conjugated with Cy3 donkey anti mouse (1:200). In order to visualize astrocytes and microglia, GFAP antibody (Sigma-Aldrich, 1:500) was added and conjugated with Cy5 donkey anti mouse (1:200). In order to detect early neurons, Doublecortin (DCX) (Cell Signaling, Boston, USA, 1:200) antibody was added and conjugated with Cy3 donkey anti rabbit (1:200). For visualization of macrophage infiltration, Iba1 antibody (WAKO, Richmond, USA 1:1000) was added and conjugated with Cy3 donkey anti rabbit (1:200). The staining Iba1 included a pretreatment: 30 min in sodium citrate 10 mM (preheated in oven) at 80°C; 20 min in the same solution at room temperature; slices were washed 3 times in KPBS for 5 min. Sections were fixed in -20°C acetone for 20 min and washed again 3 times with KPBS. The nuclei were counterstained by Hoechst 33342 (Sigma-Aldrich).

IHC images were acquired either with a fluorescent microscope (BZ-9000 Keyence, Osaka, Japan) equipped with a monochrome/color switching CCD camera or with a confocal microscope (Leica SP8X, Leica, Wetzler, Germany).

### Statistical analysis

Data were analyzed using a Mann-Whitney U-test. A p value lower than 0.05 was considered significant. SPSS (IBM, v.20) was used for statistical inferences.

## Results

### Generation of transgenic and labeled hNSCs

We have generated a transgenic hNSC line constitutively expressing two imaging reporters, i.e. the green fluoresent protein copGFP (GFP) to trace the cells in histology, and the firefly luciferase 2 (Luc2) for BLI of viable cells. The imaging reporters (Fig 1A) are under the control of the EF1α promoter, shown to be suitable for stable overexpression of proteins especially in stem cells [25]. The transduced H9-EF1-Luc2-GFP cells strongly express GFP, detectable *in vitro* by fluorescence microscopy (Fig 1B).

**Fig 2:**
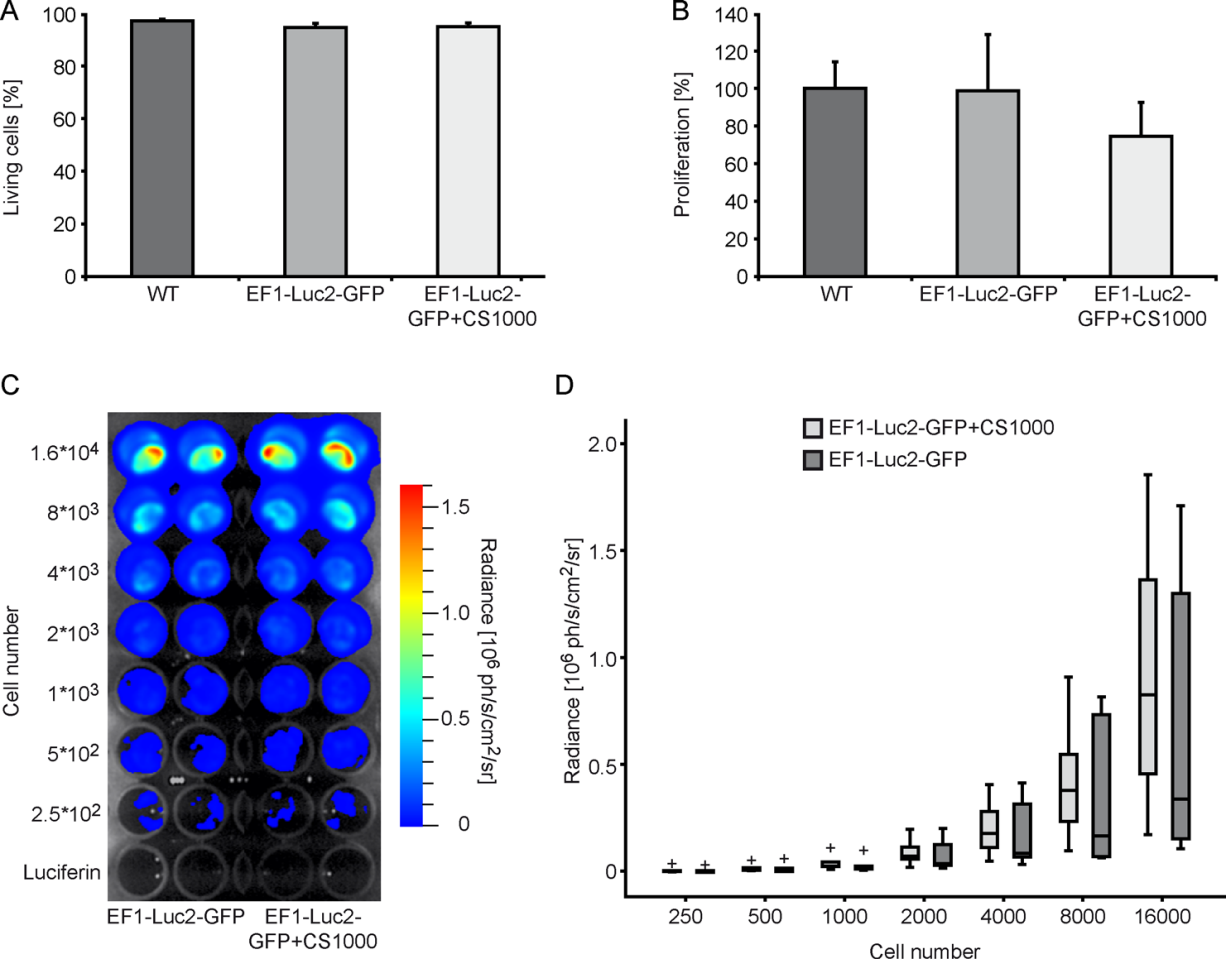
Effect of the transduction and ^19^F labeling on hNSCs. (A) Cell viability is shown for WT hNSCs and transgenic EF1-Luc2-GFP hNSCs with and without ^19^F labeling (n = 6–8). (B) Cell proliferation was compared among different cell lines. The values were normalized to the WT hNSCs and expressed in percentage (n = 5). (C) *In vitro* BLI signal from transgenic unlabeled hNSCs compared to ^19^F labeled cells. (D) *In vitro* BLI signal is displayed for a dilution series of cells (labeled and unlabeled) in 6 independent experiments. (+) outliers at least 1.5x interquartile range.

The H9-EF1-Luc2-GFP cell line was incubated with ^19^F agent for unambiguous detection by ^19^F-MRI. In order to investigate any potential negative effects of the transducing and labeling procedure on cell function, we performed *in vitro* viability and proliferation tests (Fig 2A and 2B). The transduction had only minor influence on the survival rate of the cells (survival rate decreased from 97 ± 1.4% to 95 ± 1.4%). The effect after ^19^F labeling was also only minor (survival rate decreased to 95 ± 1.4%). Transduction had no significant effect on the proliferation behavior of the cells (100 ± 14.1% in WT cells and 99 ± 29.9% in transduced cells). However, the proliferation of ^19^F labeled H9-EF1-Luc2-GFP cells decreased to 75 ± 18% (Fig 2A and 2B). In summary, the transduction and labeling had no significant effect on the cell viability, while labeling of the cells lead to minor effects on the cell proliferation.

### *In vitro* detectability and quantification of hNSCs

The efficacy of the transgenic hNSCs to generate bioluminescence signal was first evaluated *in vitro*. An example of an *in vitro* BL image acquired on a dilution series of hNSCs is shown in Fig 2C. H9-EF1-Luc2-GFP cells with (right) and without ^19^F labeling (left) are compared. BLI signal increases with the number of cells (dilution series—16, 8, 4, 2, 1, 0.5, 0.25 x 10^3^ cells). A detection threshold of less than 250 cells within 1h of acquisition was determined for both labeled and unlabeled cells (Fig 2D). In general, ^19^F labeled cells exhibit a higher median value of flux than unlabeled cells but this was not statistically significant.

In detail, for unlabeled cells, the median values were 3.43, 1.79, 0.91, 0.44, 0.21, 0.11, and 0.05*10^5^ ph/cm^2^/s/sr (from the highest to the lowest concentration). While in ^19^F labeled cells the median values were 8.28, 3.84, 1.84, 0.79, 0.31, 0.16, 0.08 *10^5^ ph/cm^2^/s/sr.

**Fig 3:**
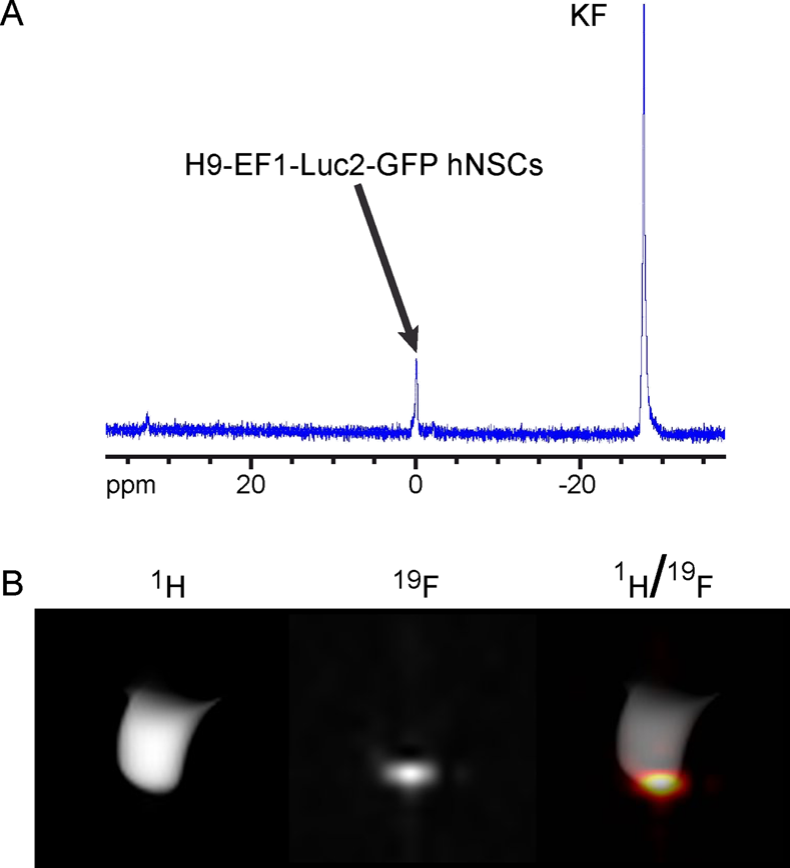
*In vitro* detectability of ^19^F labeled hNSCs by means of ^19^F MRI and ^19^F MRS. (A) ^19^F MRS of labeled hNSCs and a KF solution as internal standard to quantify the amount of ^19^F atoms per cell (B) high resolution ^1^H MR image (left), acquired during the same session of the labeled cells ^19^F MR image (center). ^1^H and ^19^F images are then merged to obtain a correct spatial localization (right).

The efficacy of ^19^F labeling of H9-EF1-Luc2-GFP cells was evaluated by means of ^19^F MRS *in vitro* (Fig 3A). ^19^F MRS allows the quantification of ^19^F atoms per cell comparing the peak generated by the cell pellet to the peak of a reference compound. The mean value of ^19^F atoms per cell was found to be 5.50*10^10^ ± 3.19*10^10^. A representative image of a cell pellet containing 2.4*10^6^ cells is displayed in Fig 3B. The total acquisition time was 3min 20s.

### Multimodal *in vivo* imaging of transplanted hNSCs

**Fig 4:**
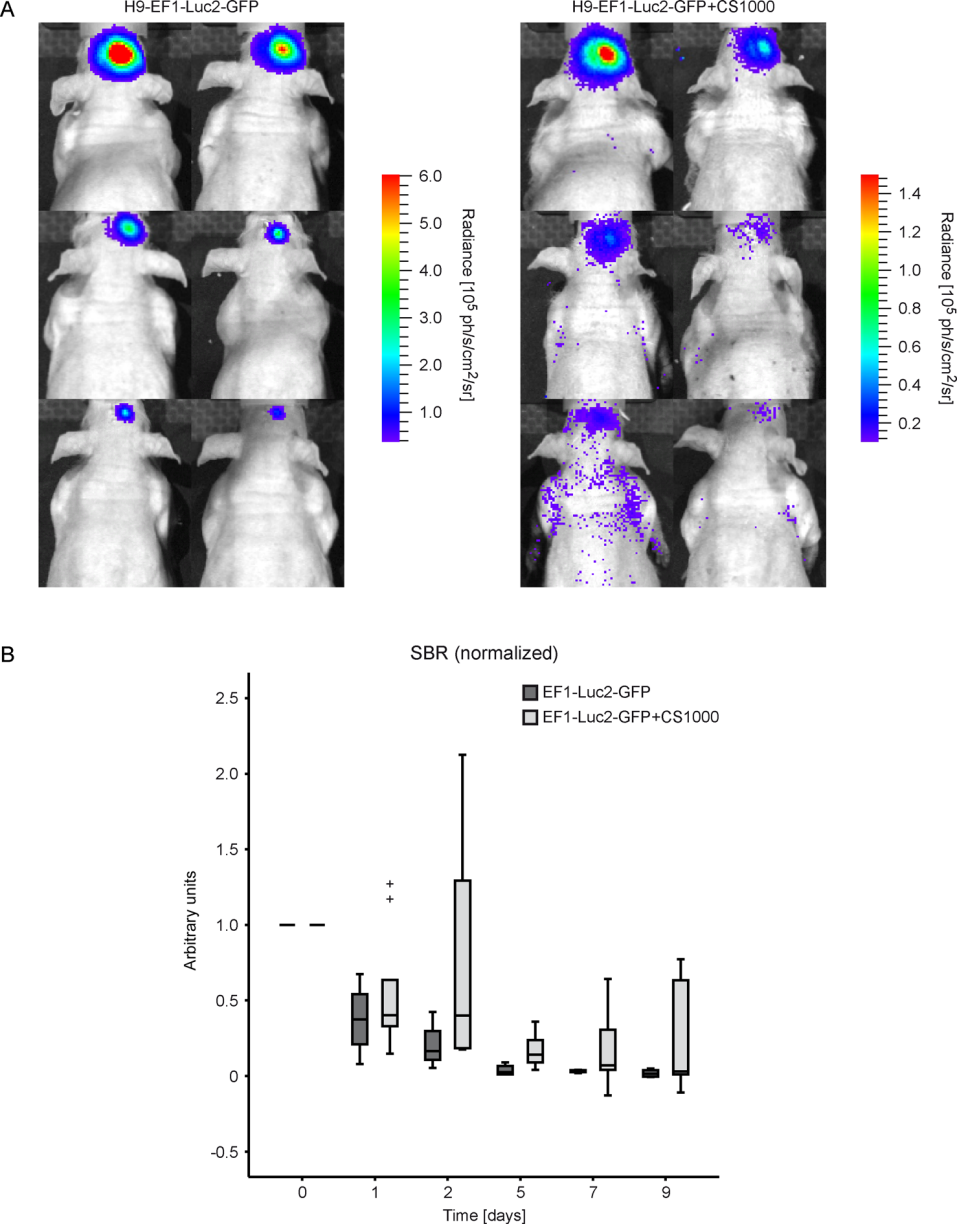
Longitudinal evaluation of cell viability by *in vivo* BLI. (A) BL images of unlabeled (left) and labeled (right) hNSCs, implanted in the right striatum and longitudinally evaluated from day 0 to day 9 post implantation [^19^F labeled cells day 0 (n = 9), day 1 (n = 9), day 2 (n = 8), day 5 (n = 8), day 7 (n = 7), day 9 (n = 5) / unlabeled cells day 0–9 (n = 4)]. (B) SBR (signal to background ratio) normalized to the first time point shows a decrease of cell viability within one week. (+) outliers at least 3x interquartile range.

In order assess the combination of ^19^F MRI and BLI for a multimodal imaging approach to track implanted stem cells in the brain in a longitudinal study, we implanted 1 x 10^5^ cells in the right striatum of mice. Cell viability was tested *in vivo* by BLI in grafted ^19^F labeled and unlabeled H9-EF1-Luc2-GFP cells from day 0 to day 9 post implantation. Both cell lines exhibited a strong BL signal during the first days post implantation, but subsequently the signal decreased till day 9 (Fig 4A). When normalizing the SBR to day 0 (Fig 4B), the SBR decreases to a median value of 40% for ^19^F labeled hNSCs and 38% for unlabeled hNSCs cells already at day 1 post implantation. After 2 days, the SBR was 40% and 17% for ^19^F labeled and unlabeled cells, respectively, at day 5 the SBR was 14% and 3%, at day 7 the SBR was 7% and 4% and at day 9 the SBR was 3% and 2%. A higher variability was observed for ^19^F labeled hNSCs. No statistical significance was found between ^19^F labeled and unlabeled cells.

**Fig 5:**
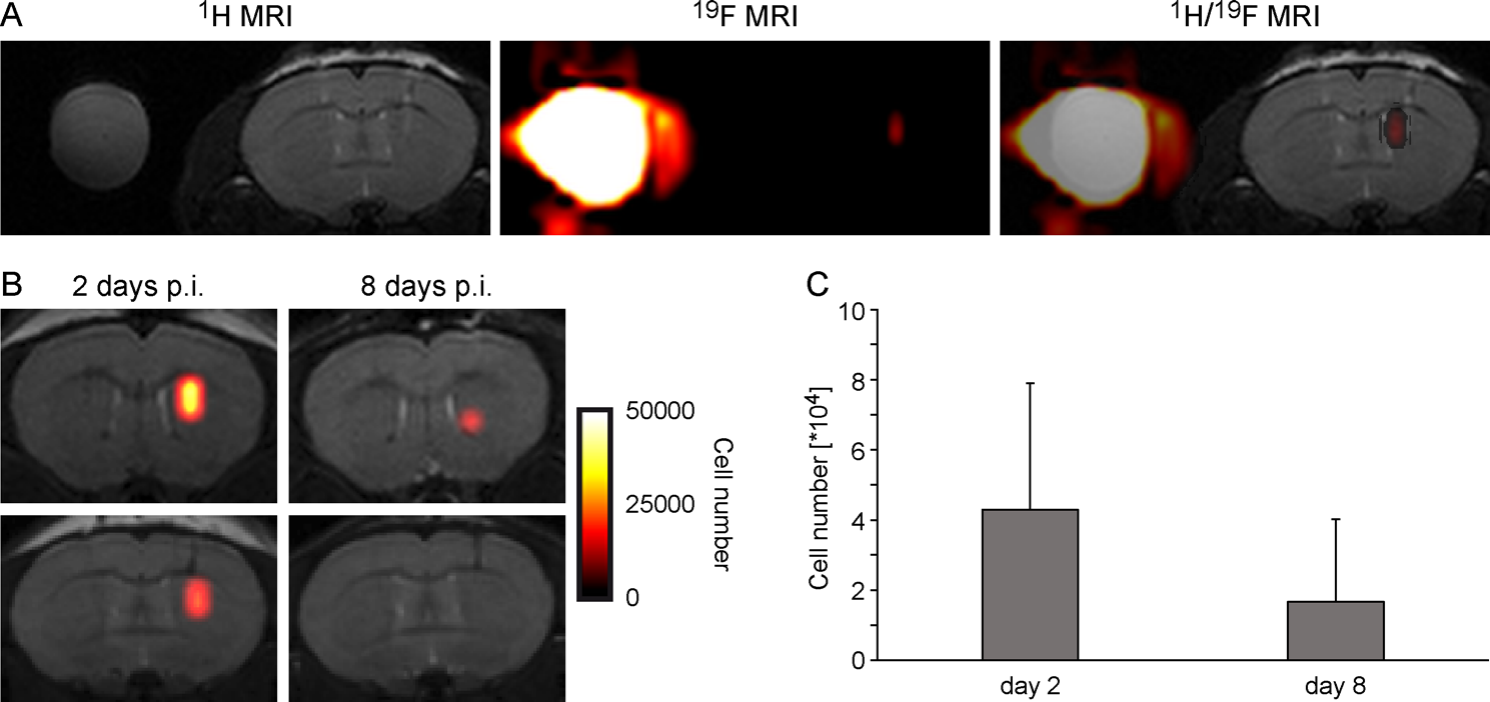
*In vivo* ^19^F MRI. (A) High resolution ^1^H MR image (left) was acquired as anatomical reference and ^19^F MR image (center) was then acquired to localize the implanted cell graft. ^1^H and ^19^F images were superimposed to combine anatomical information and spatial graft localization (right). (B) Two animals with quantitative depiction of 19F-labelled detectable cells at both two and eight days post implantation. C) Quantification of hNSCs labelled with PFPE at both, day 2 and day 8.

*In vivo* ^19^F MRI was carried out to obtain a precise anatomical localization of the hNSCs grafted in the brain (Fig 5A). By ^19^F MRI we could unambiguously show the presence of labeled H9-EF1-Luc2-GFP cells in the right hemisphere, with the ^1^H MRI providing the necessary high resolution anatomical image for localization in the striatum. The quantification of ^19^F MRI was useful to estimate the number of ^19^F cells per voxel in the brain. In our study a detection threshold of 2–3 * 10^4^ cells per voxel was estimated *in vivo* by ^19^F MRI (at an acquisition time of about 1h). In all measured mice, we detected a significant ^19^F signal from the labeled cells. Quantification of the ^19^F MRI signal indicated a decrease of ^19^F after one week post implantation. The cell number estimated by 19F MRI decreases from approximately 43.000 cells at day 2 to 17.000 cells at day 8. However, this is not statistically significant.

### Immunohistochemistry

**Fig 6:**
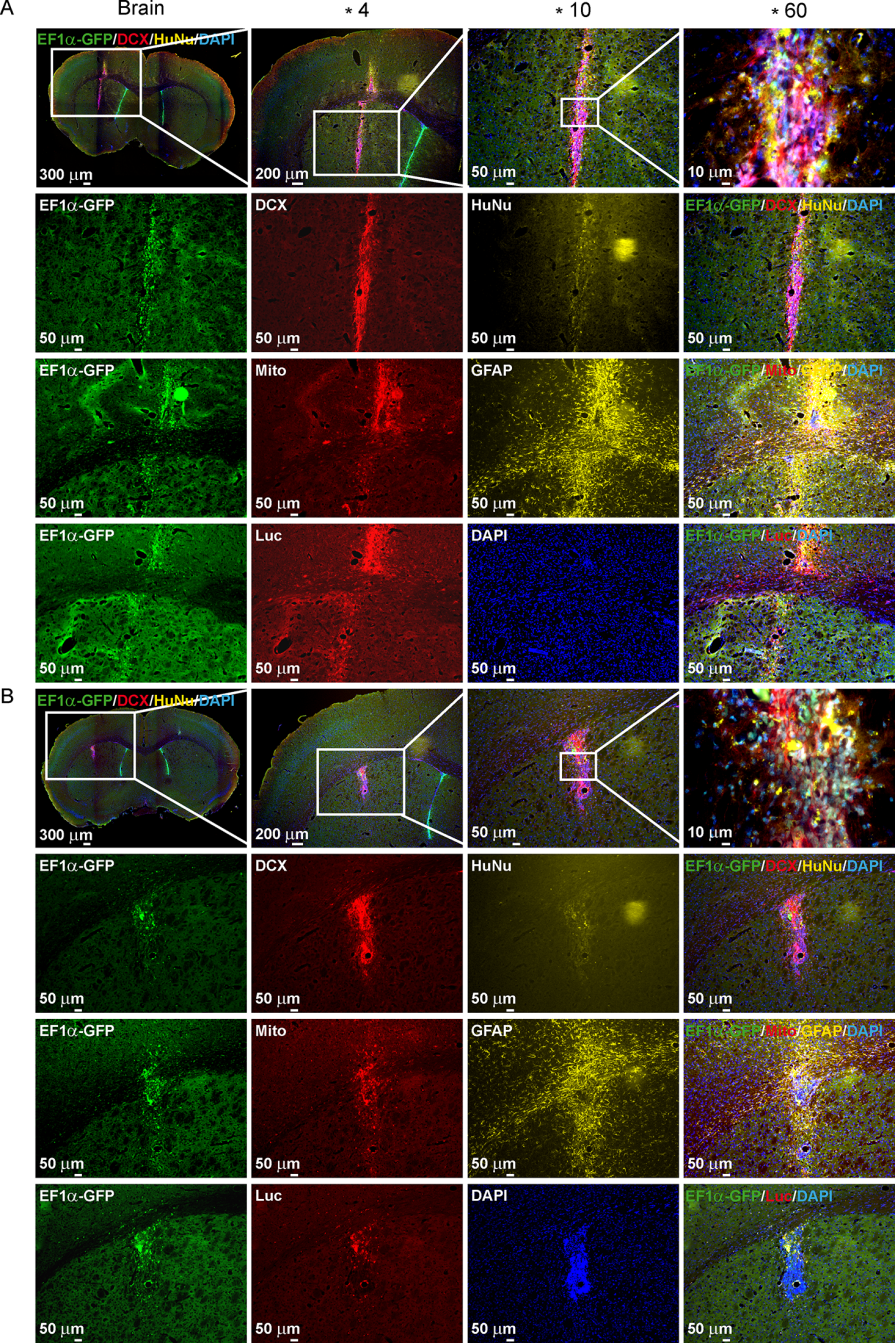
Immunohistochemistry validation of grafted hNSCS. Histology of transplanted H9-EF1-Luc2-GFP cells either labeled with ^19^F (n = 4) (A) or unlabeled (n = 4) (B) 9 days after transplantation. An overview of the mouse brain slice (scale bar: 400 μm) and higher magnification of the grafted cells verified the localization of the transplanted cells (4x magnification, scale bar: 200 μm / 10x magnification, scale bar: 50 μm / 60x magnification, scale bar: 10 μm). GFP-transgene expression (green) and immunostainings with antibodies against: DCX, neuronal marker; HuNu, human nuclei marker; Mito, human mitochondria; GFAP, astrocyte marker; Luc, luciferase marker.

At the end of the ^19^F MRI and BLI *in vivo* study, qualitative histology was performed. The labeled (Fig 6A) and unlabeled (Fig 6B) H9-EF1-Luc2-GFP cells were stained equally to compare the differentiation behavior, the reporter expression and the immune reaction of the grafted cells.

**Fig 7:**
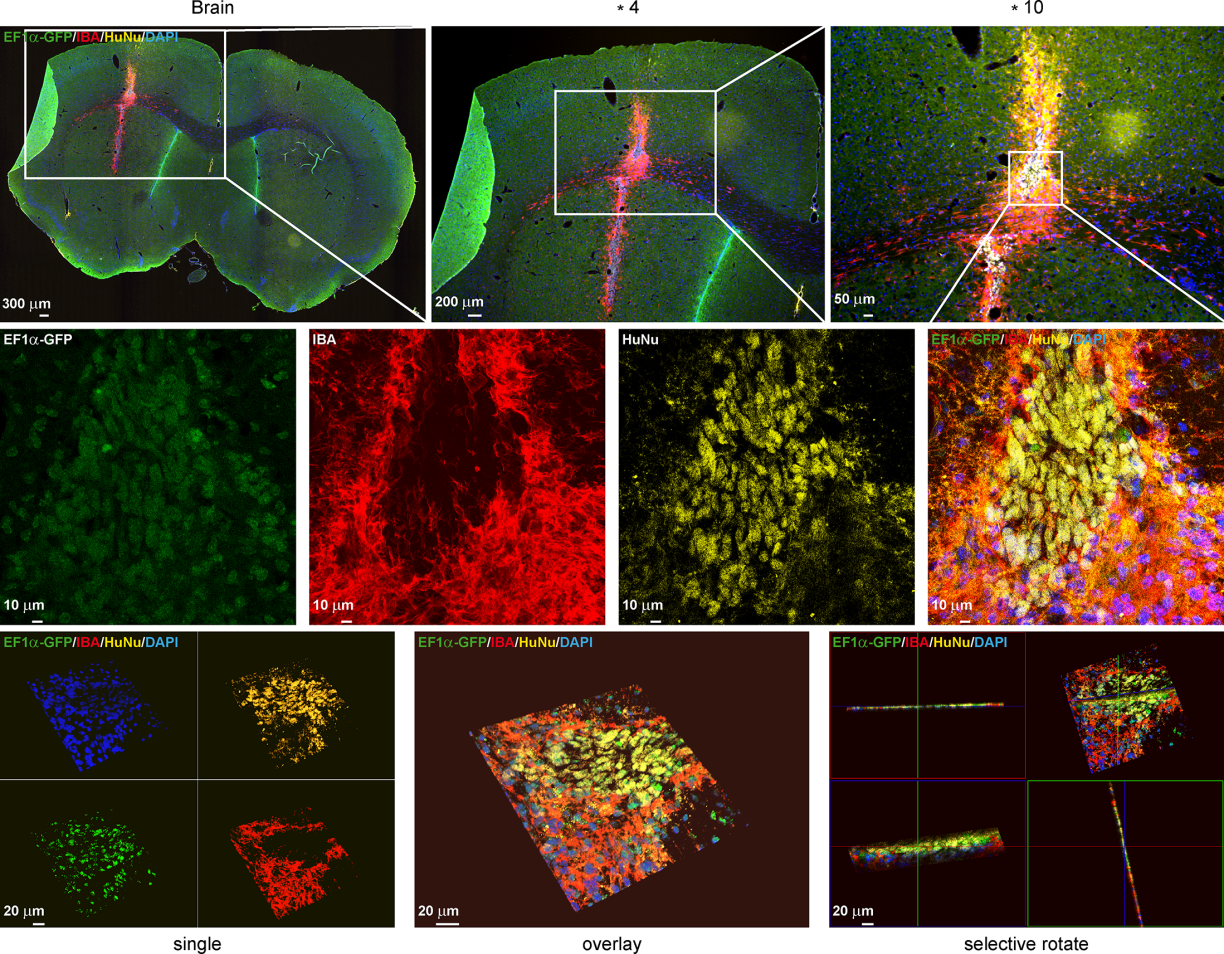
Histological analysis of graft survival and immune response of host tissue. An overview of the mouse brain slice (scale bar: 400 μm) and higher magnification confirm that Iba1 positive cells surround the cell graft (4x magnification, scale bar: 200 μm / 10x magnification, scale bar: 50 μm / 60x magnification, scale bar: 10 μm). GFP-transgene expression (green) and immunostainings with antibodies against: Iba1 (IBA), immunoreaction and HuNu, human nuclei marker. In the lower row 3D images of the IBA staining illustrate the surrounding of the cell graft by the immune cells.

An overview (Fig 6, top row and 5^th^ row) of the brain shows precisely the location of the cell graft. The endogenous GFP fluorescence well represented the grafted cells (left most column). For detailed analysis of the transplanted cells, pictures of the graft with higher magnification were produced (x4, x10 and x60; 1^st^ row of Fig 6A and 6B). To distinguish the grafted cells from the tissue, immunostaining against human-specific nuclei (HuNu; 2^nd^ and 6^th^ row) or human mitochondria (Mito; 3^rd^ and 7^th^ row) was performed. Both labeled and unlabeled cells showed strong immunoreactivity for anti-DCX clearly indicating that the cells had started to differentiate into early neurons within the nine days after transplantation into the brain (2^nd^ and 6^th^ row). In agreement with ^19^F data, no migration was detected. Endogenous GFP expression along with immunoreactivity for anti-luciferase demonstrate the strong imaging reporter expression. Furthermore, strong immunoreactivity for anti-GFAP exhibit an immune reaction surrounding the stem cell graft (3^rd^ and 7^th^ row). Labeled and unlabeled cells showed a strong immunoreactivity for anti-Luc indicating imaging reporter expression in the implanted cells (4^th^ and 8^th^ row). Anti-Iba1 staining for microglia and macrophages clearly demarcated an accumulation of immune cells surrounding the NSC graft, as noted on confocal microscopy (Fig 7).

## Discussion

### Characterisation of ^19^F labeled transgenic hNSCs *in vitro*

A new transgenic cell line for a longitudinal multimodal imaging strategy was successfully applied in this study. The strategy of combining ^19^F MRI / BLI / FLI imaging modalities allows the monitoring of cell vitality and cell localization of transgenic human NSCs.

We have shown that transduction procedures do not affect cell viability and proliferation while the ^19^F labeling procedure decreases cell proliferation. This finding is in agreement with previous research on SPIO labeling [26, 27] and ^19^F labeling effects on stem cells [11, 28]. However, labeling of mesenchymal stem cells with either SPIO or 19F came to a different result; in this study no difference in viability, cell size, colony formation, labeling efficiency and also doubling time was observed [29]. Our *in vitro* BLI experiments confirmed that after transduction hNSCs luciferase was overexpressed and a threshold of 250 cells was detected on the plate-reader within 1 hour acquisition time. Furthermore, we found that the ^19^F labeling procedure has an extremely low toxicity effect on the cells and no impact was observed referring the BL signal *in vitro*.

### *In vivo* multimodal imaging of transplanted hNSCs

We have applied the recently optimized ^19^F MRI technique permitting unambiguous graft location at high sensitivity on high-resolution structural MR images [11]. *In vitro* ^19^F MRS on labeled hNSCs has confirmed the efficacy of the labeling procedure. An amount of about 10^10^−10^11 19^F atoms per cell was detected. With *in vivo* ^19^F MRI, a detectability threshold of around 10^4^ cells *in vivo* per voxel was determined. This result is in good agreement with previous studies reporting a threshold of only few thousand cells [11, 30]. Cell number in the graft was quantified by ^19^F MRI, and a reduction of stem cells in the graft was noted 1 week after transplantation, in agreement with our *in vivo* BLI findings (cf below).

Transgenic hNSCs were transplanted deep in the striatum of mouse brain and *in vivo* BLI was successfully performed to assess cell viability in a longitudinal study. Despite the heavy absorption and scattering from the thick tissue layers between the bioluminescent graft and the CCD camera, BLI signal intensity was intense after implantation. However, shortly after grafting, the survival of transplanted H9-EF1-Luc2-GFP cells is heavily decreased during the following nine days. At day 9 post grafting, the number of viable stem cells was down to approximately 5%. However, no significant difference in viability was observed between ^19^F labeled and unlabeled stem cells, indicating that the ^19^F labeling is well tolerated by the stem cells. This is in good agreement with the *in vitro* BLI evidence. These findings are also in line with previous work showing the survival rate of grafted NSCs into healthy or inflamed brain to be less than 2% after approximately two weeks [15, 28, 31]. It must be noted that in those earlier studies, all cells were of murine origin while the present cells are a human NPC cell line. In all these past and present studies, cell survival decreased rapidly after implantation into subcortical tissue, i.e. corpus callosum [15] or striatum [28, 31]. In contrast, we have recently implanted the same NPC cell line, used in the present study, into the cortex of the same healthy mouse strain [16] and observed no significant change in viability over several weeks. The viability of these NPCs over time depending on their implantation location is compared in S1 Fig. Although the cortical and striatal implantation experiments were independent studies, our findings propose that the supporting milieu has an effect on graft survival [32]., Moreover, it has been reported that the transplantation site within the brain is crucial for graft survival. In this context, a possible explanation for NSC survival or death could be different levels of neurotrophin factors in different brain regions. Although we provide no direct proof in our study that BDNF is a key player in graft vitality, we want to emphasize that BDNF promotes cell survival and being of much stronger expression in the cortex than in the striatum [33–35].

Immunohistochemical stainings of the grafted human NSCs completed the multimodal imaging approach. A wide range of stainings was performed in order to characterize and localize the cells in the brain. Endogenous GFP served as marker to trace the implanted cells back in histology. Furthermore, the presence of human NSCs was confirmed by the HuNu^+^ or Mito^+^ staining. An overview of the brain and higher magnifications of the grafted cells showed that the surviving transplanted cells differentiate to early neurons, shown by a strong immunoreactivity for anti-DCX. Moreover, we verified by higher magnification triple positive cells–DCX+/GFP+/HuNu+—indicating early neuronal differentiation of implanted hNSCs (S2 Fig). No difference in differentiation behavior or marker expression was seen in labeled or unlabeled ^19^F grafted cells. GFAP^+^ staining highlighted the presence of astrocytes along the injection site. A strong immunoreactivity for Iba1^+^ cells indicates the presence of microglia and macrophages which enclose the grafted cells. We therefore hypothesize, that the massive cell loss within one week after grafting is due to the severe inflammatory response. It has been reported that the grafting of cells in the CNS results in significant disruption of the CNS architecture and substantial cell graft mortality [36]. Moreover, there are indications that the evoked immune responses following NSC implantation create a pro-inflammatory environment hence decrease the possibility of long-term survival of transplanted NSC [37, 38]. But is encouraging that the fraction of cells surviving in the particular milieu of the striatum already start to differentiate into early neurons within the short observation period. These graft derived neurons may replace dead striatal neurons in a therapeutic approach for the treatment of diseases which affect primarily the neurons in the striatum (for example in Huntington’s disease).

## Conclusions

A new tool for multimodal imaging was presented for *in vivo* applications of transgenic hNSCs in deep brain grafting. The combination of BLI and ^19^F MRI is advantageous as it provides simultaneous functional and anatomical information about the cell fate. Our results indicate that despite a major loss of viable cells shortly after grafting, NSCs in the striatum start to differentiate into neurons making them potential players for a cell replacement therapy.

## Supporting Information

S1 Fig Comparison of the survival ratio of transplanted cortical and striatal hNSCs. Quantitative *in vivo* BL imaging of H9-EF1-Luc2-GFP cells transplanted either in cortex (black lane; n = 4) or in striatum (gray lane; n = 4) of nude mice. Distinct decrease of bioluminescence signal in cells transplanted in the striatum was observed over time (BLI measurement at day 0, 1, 2, 5, 7 and 9). No significant decrease was detected in the cortex transplanted hNSC (BLI measurement at day 0, 1, 4, 7 and 11). Luc2 signal is normalized to day 0 (each time point is divided by value at day 0). Data of cortical implantation were taken from an earlier study [16].
(PDF)

S2 Fig Histological analysis of differentiation behavior of grafted H9-EF1-Luc2-GFP cells nine days after transplantation. Cells were either labeled with 19F (n = 4) (A) or unlabeled (n = 4) (B). GFP-transgene expression (green) and immunostainings with antibodies against: DCX, neuronal marker, and HuNu, human nuclei marker (60x magnification; scale bar: 10μm).
(PDF)
