# Supplementary figures and images for: In Vivo Fate Imaging of Intracerebral Stem Cell Grafts in Mouse Brain
Source: PLoS One. 2015 Dec 7;10(12):e0144262. doi: 10.1371/journal.pone.0144262 (PMC4671578; doi:10.1371/journal.pone.0144262)

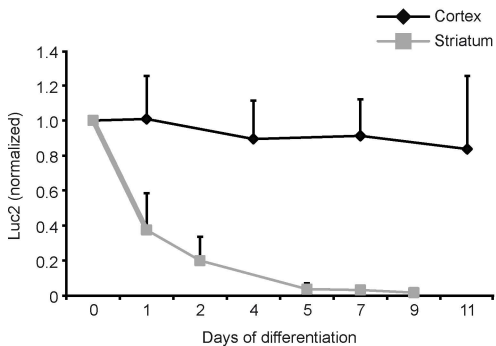

Supplement: S1 Fig — Quantitative in vivo BL imaging of H9-EF1-Luc2-GFP cells transplanted either in cortex (black lane; n = 4) or in striatum (gray lane; n = 4) of nude mice. Distinct decrease of bioluminescence signal in cells transplanted in the striatum was observed over time (BLI measurement at day 0, 1, 2, 5, 7 and 9). No significant decrease was detected in the cortex transplanted hNSC (BLI measurement at day 0, 1, 4, 7 and 11). Luc2 signal is normalized to day 0 (each time point is divided by value at day 0). Data of cortical implantation were taken from an earlier study [16]. (PDF) [file pone.0144262.s001.pdf]

**A**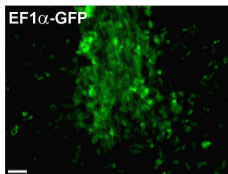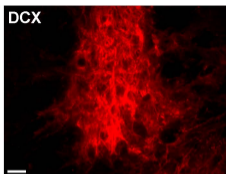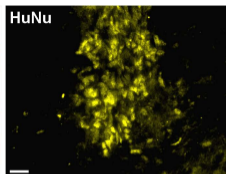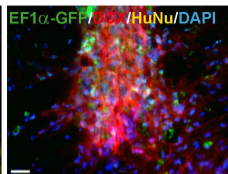**B**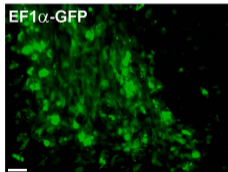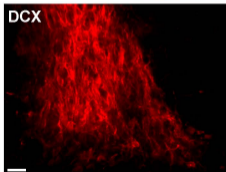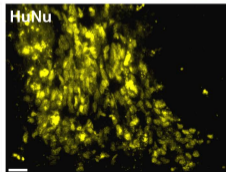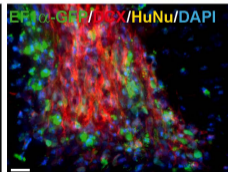

Supplement: S2 Fig — Cells were either labeled with 19F (n = 4) (A) or unlabeled (n = 4) (B). GFP-transgene expression (green) and immunostainings with antibodies against: DCX, neuronal marker, and HuNu, human nuclei marker (60x magnification; scale bar: 10μm). (PDF) [file pone.0144262.s002.pdf]
